# EphA2 affects the sensitivity of oxaliplatin by inducing EMT in oxaliplatin-resistant gastric cancer cells

**DOI:** 10.18632/oncotarget.18208

**Published:** 2017-05-24

**Authors:** Qiaocheng Wen, Zihua Chen, Zhikang Chen, Jinxiang Chen, Ran Wang, Changhao Huang, Weijie Yuan

**Affiliations:** ^1^ General Surgery, Xiangya Hospital, Central South University, Changsha, China

**Keywords:** EphA2, gastric cancer, epithelial–mesenchymal transition, oxaliplatin, drug resistance

## Abstract

Erythropoietin-producing hepatocellular receptor A2 (EphA2) is upregulated in gastric cancer tissues and cells, which is accompanied by epithelial–mesenchymal transition (EMT). The current study was designed to establish the oxaliplatin-resistant human gastric cancer cell line SGC-7901/L-OHP, to determine if EMT in these cells could be reversed, and to determine if the susceptibility of these cells to oxaliplatin was affected by silencing EphA2 expression. We found that EphA2 expression levels were upregulated in gastric cancer and associated with chemotherapy sensitivity. EphA2 and the EMT molecular markers N-cadherin and Snail were upregulated in SGC-7901/L-OHP cells, while silencing of EphA2 using small interfering RNA had the opposite effect. Moreover, silencing of EphA2 inhibited cell migration and invasion, and significantly enhanced the sensitivity of oxaliplatin-resistant gastric cancer cells to oxaliplatin. These observations demonstrate that EphA2 affects the sensitivity to oxaliplatin by inducing EMT in oxaliplatin-resistant gastric cancer cells.

## INTRODUCTION

Gastric cancer remains the third leading cause of cancer-related mortality worldwide; however, its incidence has decreased in the past six decades [1,2]. Most patients are diagnosed with this disease at an advanced stage and the 5-year survival rate for surgically resected cases ranges from 13.1% to 43.8% [3]. The incidence and mortality of gastric cancer rank second and third, respectively, among malignant tumor types in China [4]. Chemotherapy plays a decisive role in the prevention and treatment of gastric cancer recurrence and metastasis [5,6]. Oxaliplatin (L-OHP) belongs to the third generation of platinum compounds and is gradually becoming the primary drug for the treatment of advanced gastric cancer [7]. Although chemotherapeutic regimens for gastric cancer are continuously improving, adverse treatment effects following gastric cancer chemotherapy still occur in some patients. In addition, resistance to chemotherapeutic drugs occurs or develops in most tumor cells, leading to treatment failure [8–10]. Consequently, the 5-year survival rate of patients with advanced gastric cancer has not significantly increased [11–13].

The epithelial–mesenchymal transition (EMT) refers to the physiopathological process in which epithelial cell-like features are lost and mesenchymal cell-like features gradually develop in tumor cells originating from epithelia. EMT plays important roles in tumor invasion and metastasis, acquisition and maintenance of stem cell characteristics, resistance to cell death and aging, escape from immune monitoring, and drug resistance of tumor cells [14]. It is believed by many to be an early critical event in the induction of tumor invasion and metastasis. Indeed, recent studies have indicated that the occurrence of EMT in tumor cells is closely related to drug resistance [15].

The increased expression and activity of erythropoietin-producing human hepatocellular carcinoma (Eph) receptors are observed in multiple tumors and are closely related to tumor metastasis and the prognosis of patients with malignancies [16]. EphA2 is a member of the receptor tyrosine kinase (RTK) family and was the first protein shown to demonstrate tyrosine kinase activity among the Eph proteins encoded for by genes on chromosome lp36.1 [17]. Our previous studies demonstrated that EphA2 plays a key role in signal transduction pathways associated with the regulation of growth, proliferation, and metastasis of tumor cells. Conversely, silencing of EphA2 resulted in the inhibition of human gastric cancer SGC-7901 cell invasion both *in vitro* and *in vivo* [18]. However, previous studies have not determined if the EMT in oxaliplatin-resistant gastric cancer cells can be regulated by EphA2, thereby affecting associated drug resistance. The putative role of EphA2 in this phenomenon and the underlying mechanisms remain unclear and require further investigation.

In this study, the expression of EphA2 in cancer tissues and adjacent normal gastric mucosa was determined by immunohistochemistry in 120 patients with advanced gastric cancer. The chemotherapy response rate of all patients was used to analyze the association between EphA2 expression and chemosensitivity. We also used *in vitro* assays to evaluate the antitumor efficacy of oxaliplatin. The sensitivity of gastric cancer cells to oxaliplatin following silencing of EphA2 was determined using the oxaliplatin-resistant gastric cancer cell line, SGC-7901/L-OHP. The expression of EphA2 and the EMT markers, N-cadherin, Snail, and E-cadherin, were also analyzed *in vitro* by real-time quantitative polymerase chain reaction (PCR), Western blotting, and immunofluorescence analyses of the SGC-7901/L-OHP cells. In addition, cell migration and cell invasion were also studied.

## RESULTS

### EphA2 expression is associated with the therapeutic effects of oxaliplatin-based chemotherapy in patients with advanced gastric cancer

**Figure 1:**
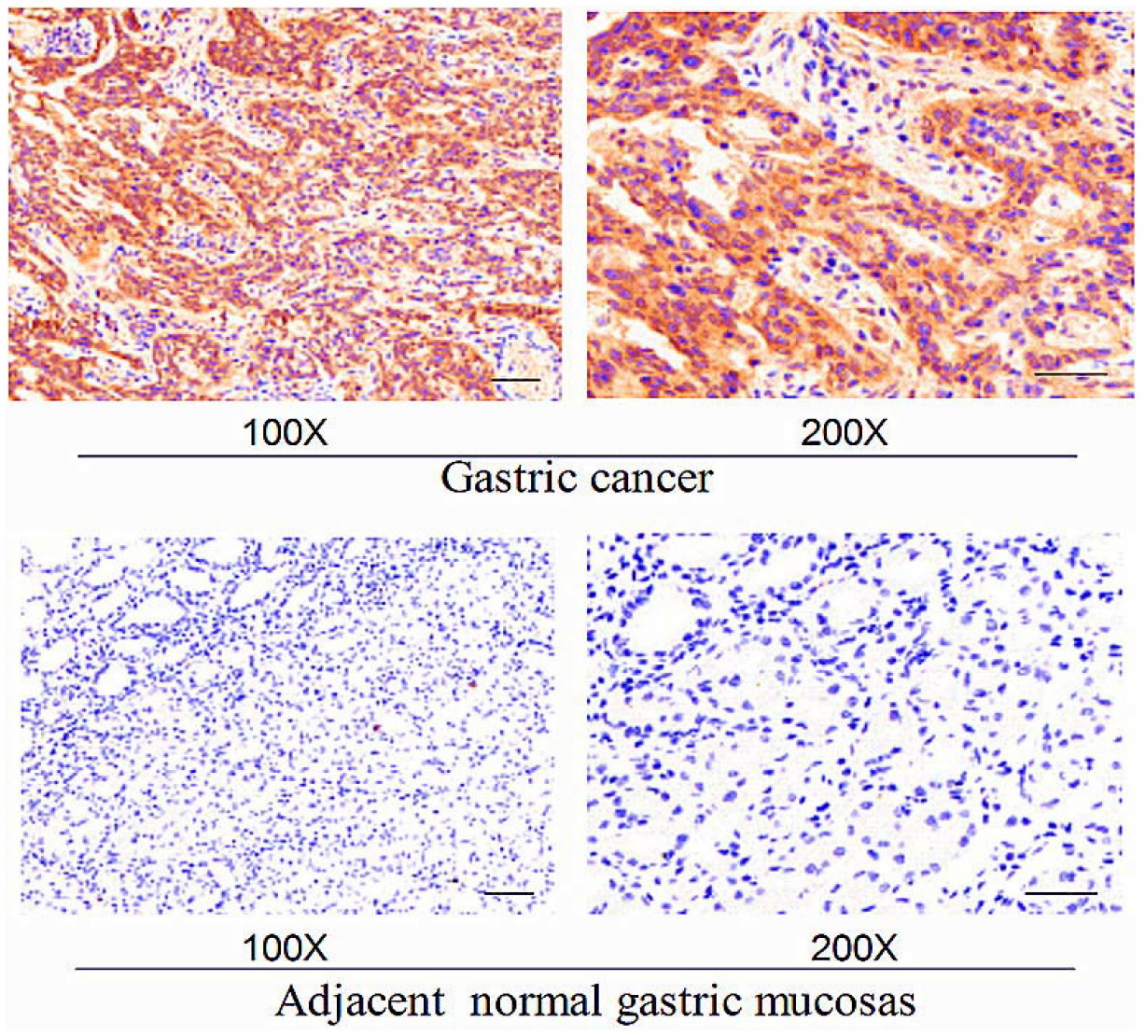
Representative expression levels of EphA2 in gastric cancer and adjacent normal gastric mucosa following immunohistochemisty Bars, 100 μm.

The expression of EphA2 in cancer tissues and adjacent normal gastric mucosa was analyzed in 120 patients with advanced gastric cancer using immunohistochemistry. Patients were treated with a 2 h continuous infusion of oxaliplatin (100 mg/m^2^) on day 1. The patients were also administered calcium folinate (400 mg/m^2^) followed by fluorouracil(5-FU, 400 mg/m^2^) for 46 h by continuous infusion of 2400 mg/m^2^ on days 1 and 2. Treatment was repeated every 2 weeks. After three of these treatment regimens, the chemotherapy response rate of all patients was analyzed to investigate the association between EphA2 expression and chemosensitivity. EphA2 showed significantly higher expression in gastric cancer tissues relative to adjacent normal gastric mucosa (Figure 1). As shown in Tables 1 and 2, the expression of EphA2 in gastric cancer tissues was significantly higher than that in adjacent normal gastric mucosa tissues (*P* < 0.05). All 120 patients with advanced gastric cancer received three cycles of FOLFOX6 chemotherapy, and the efficacy evaluation revealed complete remission (CR) in 10 cases, partial remission (PR) in 52 cases, stable disease (SD) in 41 cases, and progressive disease (PD) in 17 cases. The chemotherapy response rate (RR) was 51.67%. The RR was 78.72% and 34.24% in the EphA2-negative and Eph-A2-positive expression groups, respectively. The chemotherapy RR in the EphA2-negative expression group was higher than that in the EphA2-positive group, with a statistically significant difference (*P* < 0.05) (Table 3). Various clinical and pathological features that may affect the efficacy of chemotherapy are summarized in Table 3. Following the analysis of these features, we observed that the pathological type and low protein expression of EphA2 affected the efficacy of chemotherapy (*P* < 0.05).

**Table 1:** Expression of EphA2 in 251 cases of gastric cancer and adjacent normal gastric mucosa tissues (χ^2^ test)

Protein	Gastric cancer tissues	Gastric normal mucosa tissues	P-value
EphA2			
Positive	73	17	0.000
Negative	47	103	

Eph: erythropoietin-producing human hepatocellular carcinoma.

**Table 2:** Correlation between EphA2 and clinicopathologic features in 120 gastric cancer cases (χ^2^ test)

Parameters	n	EphA2		
		+	−	P-value
Age (years)				
<60	32	21	11	0.056
≥60	88	52	36	
Gender				
Male	61	39	22	0.314
Female	59	34	25	
Size of tumor				
<5 cm	64	31	33	0.061
>5 cm	56	42	14	
ECOG score				
0-1	58	34	24	0.219
2	62	39	23	
Histologic type				
Well and moderate	44	14	30	0.018
Poor and undifferentiated	76	59	17	
CEA (ng/mL)				
<5	91	55	36	0.102
≥5	29	18	11	

ECOG: Eastern Cooperative Oncology Group; CEA: carcino-embryonic antigen.

**Table 3:** Relationship among chemotherapy efficacy, clinicopathological features, and EphA2 expression (χ^2^ test)

Parameters	n	CR+PR	Response rate (%)	P-value
Age (years)				
<60	32	13	59.37	0.172
≥60	88	49	55.68	
Gender				
male	61	30	49.18	0.144
Female	59	32	54.24	
Size of tumor				
<5 cm	64	37	57.81	0.115
>5 cm	56	25	44.64	
ECOG score				
0-1	58	36	62.07	0.100
2	62	26	41.94	
Histologic type				
Well and moderate	44	32	72.72	0.032
Poor and undifferentiated	76	30	39.47	
CEA (ng/mL)				
<5	29	20	68.96	0.081
≥5	91	42	46.15	
EphA2				
+	73	25	34.24	0.007
-	47	37	78.72	

ECOG: Eastern Cooperative Oncology Group; CEA: carcino-embryonic antigen; Eph: erythropoietin-producing human hepatocellular carcinoma; CR: complete remission; PR: partial remission.

### Establishment of the oxaliplatin-resistant gastric cancer cell line and evaluation of its biological morphological characteristics and sensitivity to oxaliplatin

**Figure 2:**
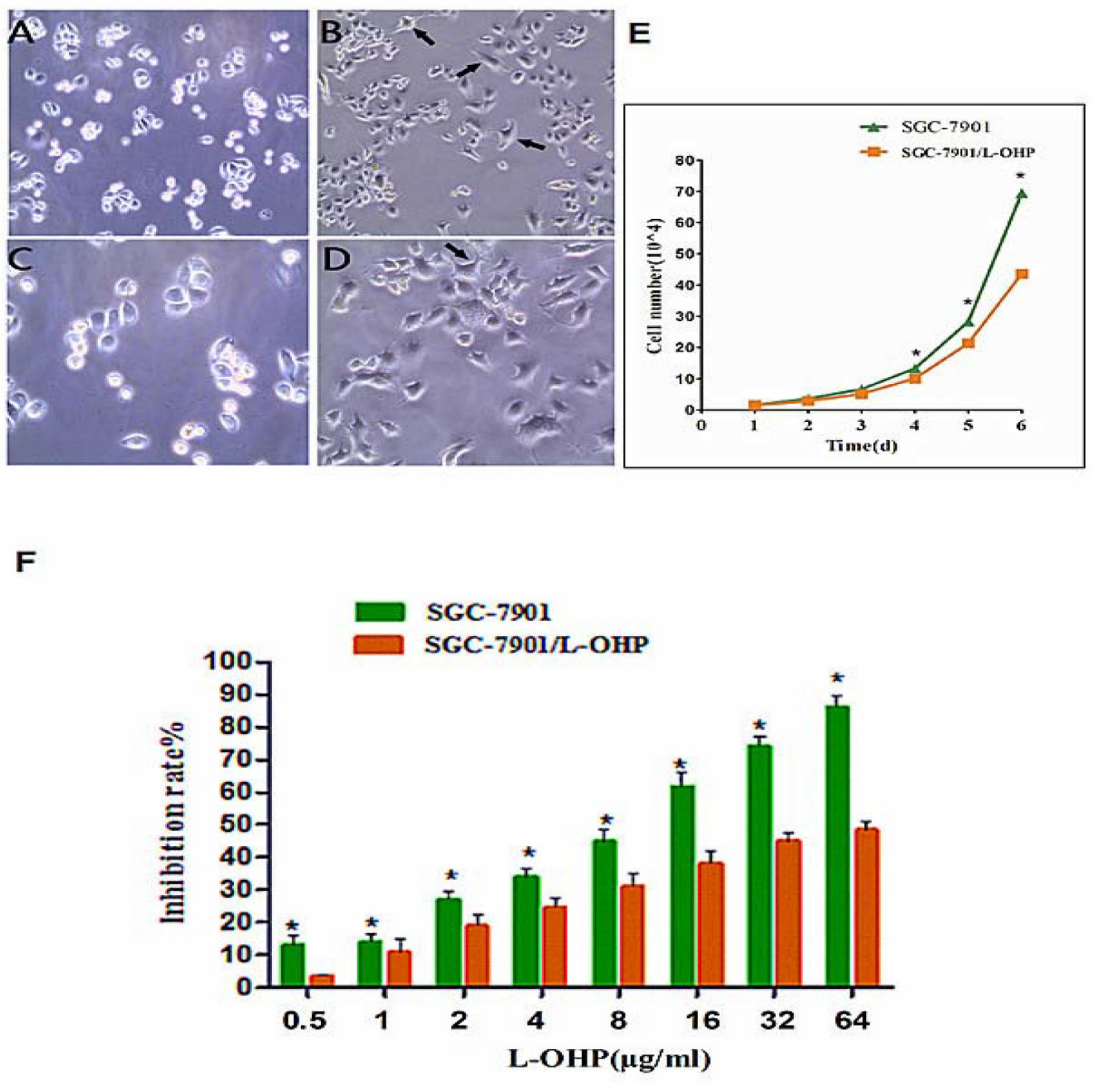
Cell morphological characteristics observed using phase-contrast micrographs **(A)** SGC-7901cells (100×). **(B)** SGC-7901/L-OHP cells (100×). **(C)** SGC-7901 cells (200×). **(D)** SGC-7901/L-OHP cells (200×, black arrows refer to mesenchymal-like phenotype cells; these cells exhibited loss of epithelial characteristics and acquisition of mesenchymal properties). **(E)** Growth curves of SGC-7901 and SGC-7901/L-OHP cells (*P < 0.05). **(F)** Inhibition rate of SGC-7901 and SGC-7901/L-OHP cells following exposure to different concentrations of L-OHP, as measured using the MTT assay. The inhibition ratio of L-OHP to SGC-7901 was significantly higher than that of L-OHP to SGC-7901/L-OHP (*P < 0.05).

Analysis of the human gastric cancer cells SGC-7901 and the oxaliplatin-resistant human gastric cancer cells SGC-7901/L-OHP under an inverted phase contrast microscope showed that the epithelial mucosa in the SGC-7901 cells was uniformly circular. On the other hand, a mesenchymal-like phenotype was observed for SGC-7901/L-OHP cells, which displayed irregular shapes such as long strip, fusiform, and polygon. The resultant morphological changes indicated that SGC-7901/L-OHP cells possessed a mesenchymal-like phenotype (Figure 2A–2D). A cell growth curve analysis revealed that compared with the gastric cancer cell line SGC-7901, the proliferation rate of the oxaliplatin-resistant gastric cancer cell line SGC-7901/L-OHP was significantly reduced (Figure 2E). The population doubling time of the SGC-7901/L-OHP cells was 27.44 ± 2.01 h, whereas that of the SGC-7901 cells was 23.89 ± 1.56 h, with a statistically significant difference (*P* < 0.05). These results suggest that the oxaliplatin-resistant gastric cancer cell line SGC-7901/L-OHP exhibited reduced proliferative capacity. The resistance level of SGC-7901/L-OHP cells to L-OHP was determined using the MTT (3- (4,5-dimethythiazol-2-yl)-2,5-diphenyl tetrazolium bromide) assay. The results indicated that the inhibition ratio of L-OHP to SGC-7901 gradually increased, whereas the inhibition ratio of L-OHP to SGC-7901/L-OHP was significantly lower at an identical concentration of L-OPH (*P*<0.05) (Figure 2F). The IC_50_ values within 48 h of treatment of SGC-7901/L-OHP and SGC-7901 cells were calculated as 97.90 and 10.10 μg/mL, respectively, following probit regression analysis. The resistance index (RI) of SGC-7901/L-OHP cells to L-OHP was calculated to be 9.7 using the following formula: RI = IC_50_ (drug resistance cells)/IC_50_ (parental cells). These results suggest that there is increased resistance of oxaliplatin-resistant gastric cancer cells to L-OHP.

### Expression of EphA2 in SGC-7901 and SGC-7901/L-OHP cells

**Figure 3:**
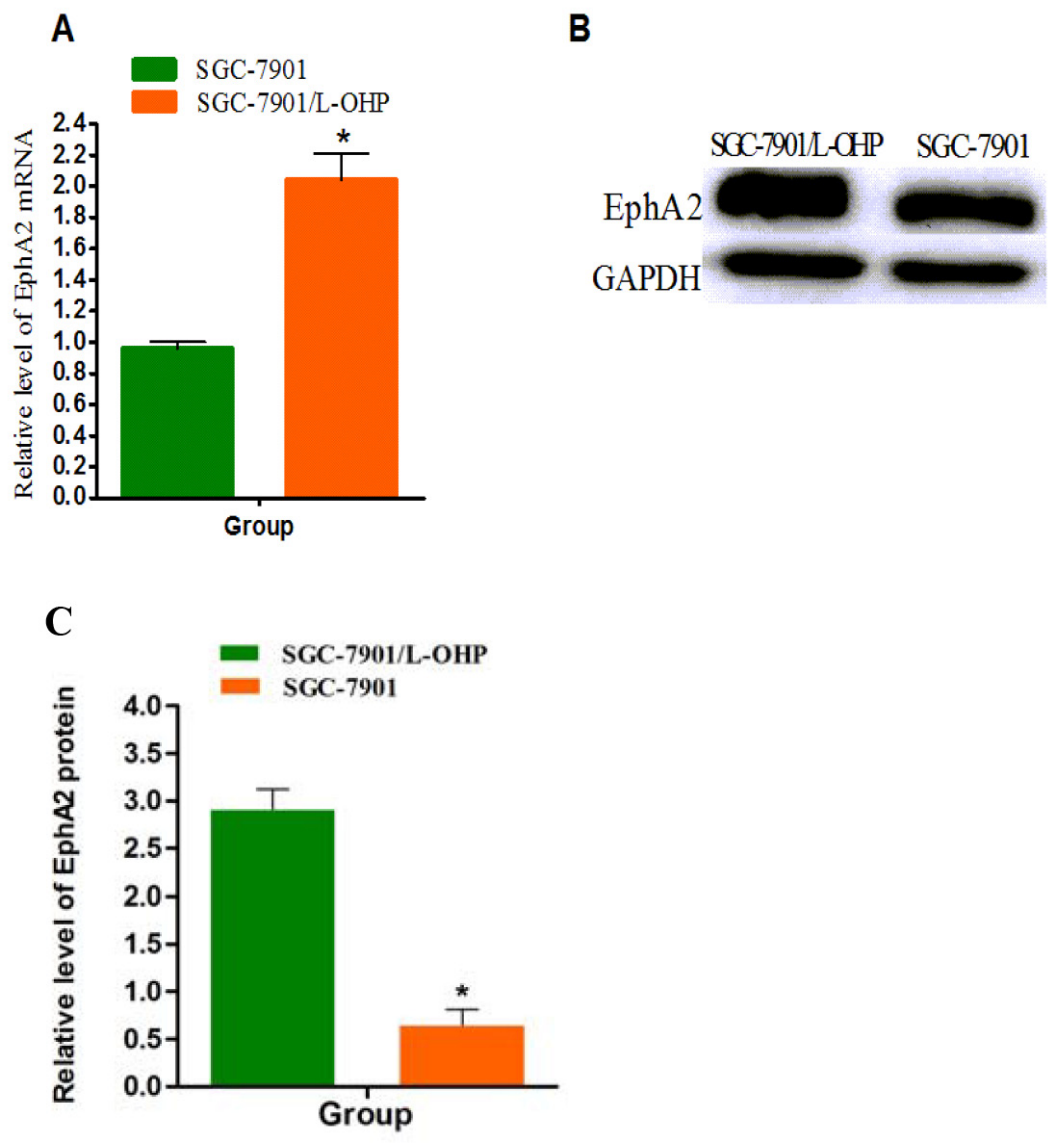
EphA2 overexpression in SGC-7901/-L-OHP cells **(A)** mRNA expression levels of EphA2 in SGC-7901 and SGC-7901/-L-OHP cells were assayed by quantitative real-time PCR. 18S rRNA was used as an internal control (ctrl). Compared with ‘ 18S rRNA group (*P < 0.05). **(B and C)** Protein levels of EphA2 in different groups of cells were assayed by Western blotting. GAPDH was used as the internal control. Relative accumulation of proteins in the SGC-7901/-L-OHP group compared with the SGC-7901 group is indicated (*P < 0.05).

The mRNA and protein expression of EphA2 in SGC-7901 and SGC-7901/L-OHP cells was analyzed by real-time qPCR and Western blotting, respectively. The relative expression of EphA2 mRNA and protein in SGC-7901 was set to 1. The results indicated that the relative expression of EphA2 mRNA and protein in SGC-7901/L-OHP cells was significantly increased compared to expression in SGC-7901 cells (*P* < 0.05) (Figure 3A–3C).

### Effects of EphA2 knockdown on oxaliplatin-resistant gastric cancer cells

**Figure 4:**
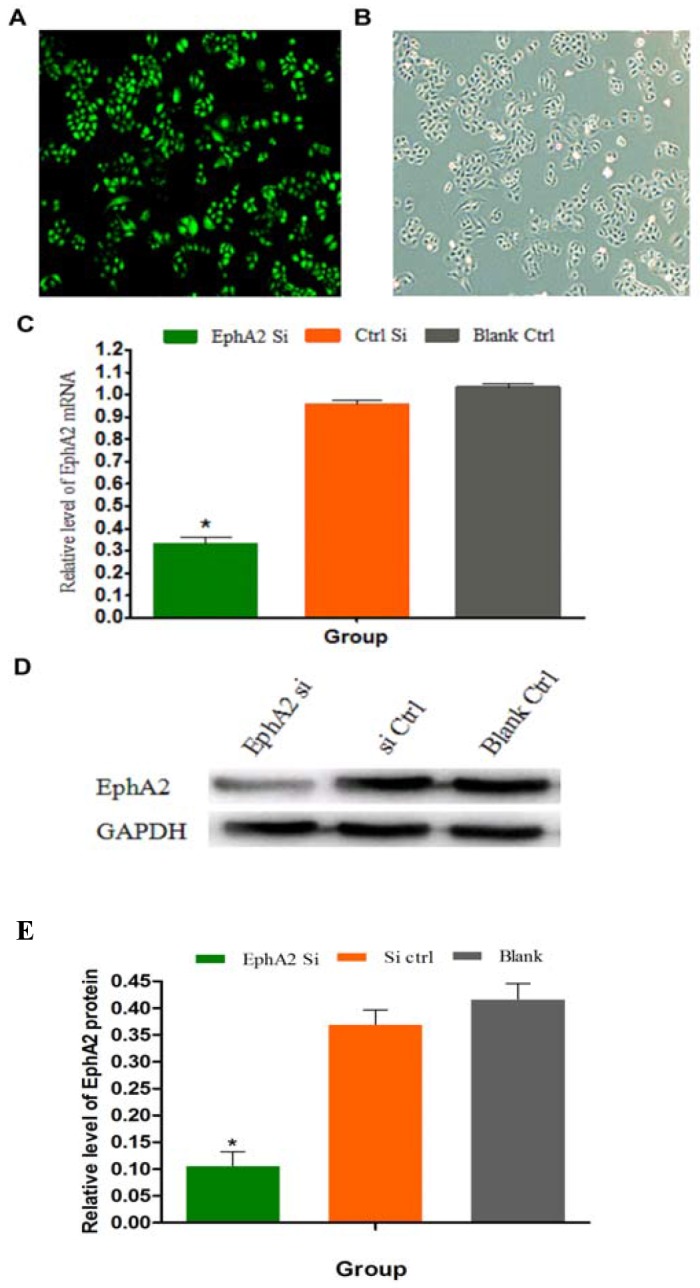
SGC-7901/L-OHP cells transfected with EphA2 siRNA; EphA2 mRNA and protein expression in the SGC-7901/L-OHP cells was silenced **(A)** SGC-7901/L-OHP cells visualized by fluorescence light microscopy (200×). **(B)** SGC-7901/L-OHP cells visualized using an ordinary light microscope (200×). **(C)** The mRNA expression of EphA2 in SGC-7901/L-OHP cells was assayed by quantitative real-time PCR. 18S rRNA was used as the internal control (ctrl). Compared with ‘the blank’ group (* *P* < 0.05). **(D and E)** EphA2 protein expression in SGC-7901/L-OHP cells was assayed by Western blotting; GAPDH was used as the internal control. The relative accumulation of proteins in the cells compared with the ‘blank ctrl’ group is indicated (*P < 0.05).

The oxaliplatin-resistant gastric cancer cell line SGC-7901/L-OHP was transfected with EphA2 siRNA using Lipofectamine 2000. Insignificant morphological changes of these cells before and after transfection were observed under an inverted fluorescence microscope, and the transfection efficiency was > 80% (Figure 4A and 4B); this efficiency was suitable to analyze the effects of siRNA. The mRNA and protein expression of EphA2 in SGC-7901/L-OHP cells was significantly higher than that in SGC-7901 cells. At 48 h post-transfection of SGC-7901/L-OHP cells with EphA2 siRNA, the mRNA and protein expression levels of EphA2 were evaluated by quantitative real-time PCR and Western blotting, respectively. Compared with the control cells, the mRNA and protein levels of EphA2 in the EphA2 knockdown cells significantly decreased (*P* < 0.05) (Figure 4C–4E).

### EphA2 promotes the EMT in SGC-7901/L-OHP cells

**Figure 5:**
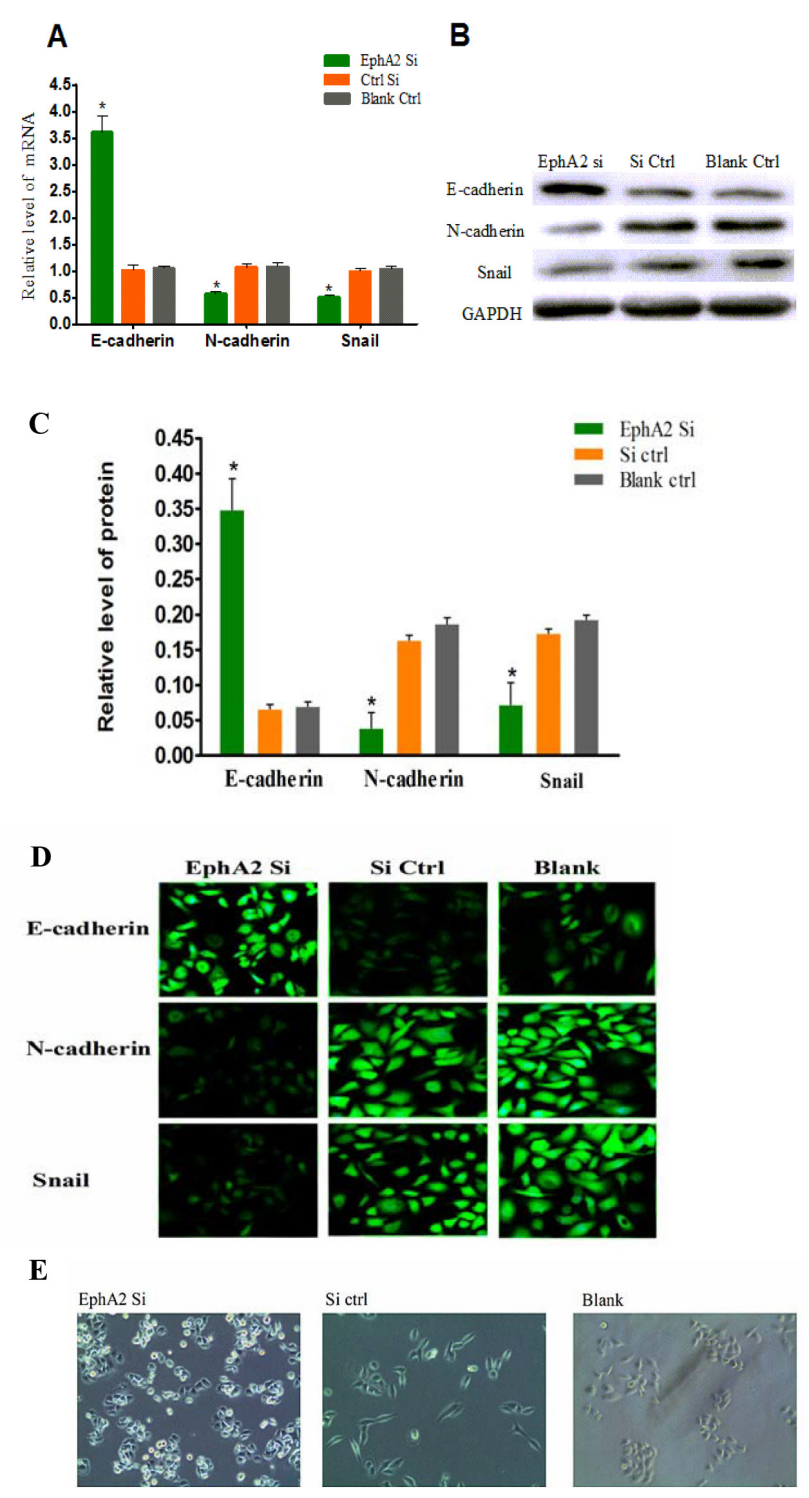
EphA2 regulate EMT markers **(A)** The mRNA expression levels of three EMT markers (E-cadherin, N-cadherin, and Snail) in SGC-7901/L-OHP cells were assayed by quantitative real-time PCR. 18S rRNA was used as the internal control (ctrl). Compared with ‘the blank’ group (*P < 0.05). **(B and C)** Protein levels of the three EMT markers in were assayed by Western blotting; GAPDH was used as the internal control (Ctrl). Relative protein accumulation of proteins in cells compared with the ‘blank ctrl’ group is indicated (*P < 0.05). **(D)** Immunofluorescence reveals the presence of three the EMT markers. Targeted proteins were stained green. **(E)** Post-transfection of SGC-7901/L-OHP cells with EphA2 siRNA. Changes in cell phenotype are shown as the loss of mesenchymal properties and acquisition of epithelial characteristics.

It is well known that E-cadherin, N-cadherin, and Snail are specific markers for the EMT process. After transfection of SGC-7901/L-OHP cells with EphA2 siRNA for 48 h, the mRNA and protein expression of N-cadherin and Snail was examined by quantitative real-time PCR and Western blotting, respectively. The results demonstrated that the expression levels of N-cadherin mRNA and protein were significantly increased (*P* < 0.05) in the EphA2 knockdown cells compared with the negative control and blank control cells (*P* < 0.05). Conversely, the expression levels of N-cadherin and Snail mRNA and protein were reduced in the EphA2 knockdown cells compared with the negative control and blank control cells (*P* < 0.05) (Figure 5A–5C). These results suggest that expression of the epithelial marker, E-cadherin, in SGC-7901/L-OHP cells was upregulated at the mRNA and protein levels, whereas expression of the mesenchymal marker, N-cadherin, and the transcription factor, Snail, was inhibited in SGC-7901/L-OHP cells following transfection with EphA2 siRNA. Similar results were obtained by immunofluorescence staining (Figure 5D). Morphological characteristics of the EMT include the loss of epithelial characteristics and the acquisition of mesenchymal properties such as spindle-like fusiform shape and loss of sheet-like architecture in epithelial cells. After transfection of SGC-7901/L-OHP cells with EphA2 siRNA, an epithelioid phenotype was visualized under an inverted phase contrast microscope. Conversely, the control and blank groups still exhibited a mesenchymal-like phenotype (Figure 5E). These results suggest that EphA2 is a driving force of EMT in SGC-7901/L-OHP cells, and silencing its expression might reverse the EMT process.

### Changes in migration and invasion ability in oxaliplatin-resistant gastric cancer cells after transfection with EphA2 siRNA

**Figure 6:**
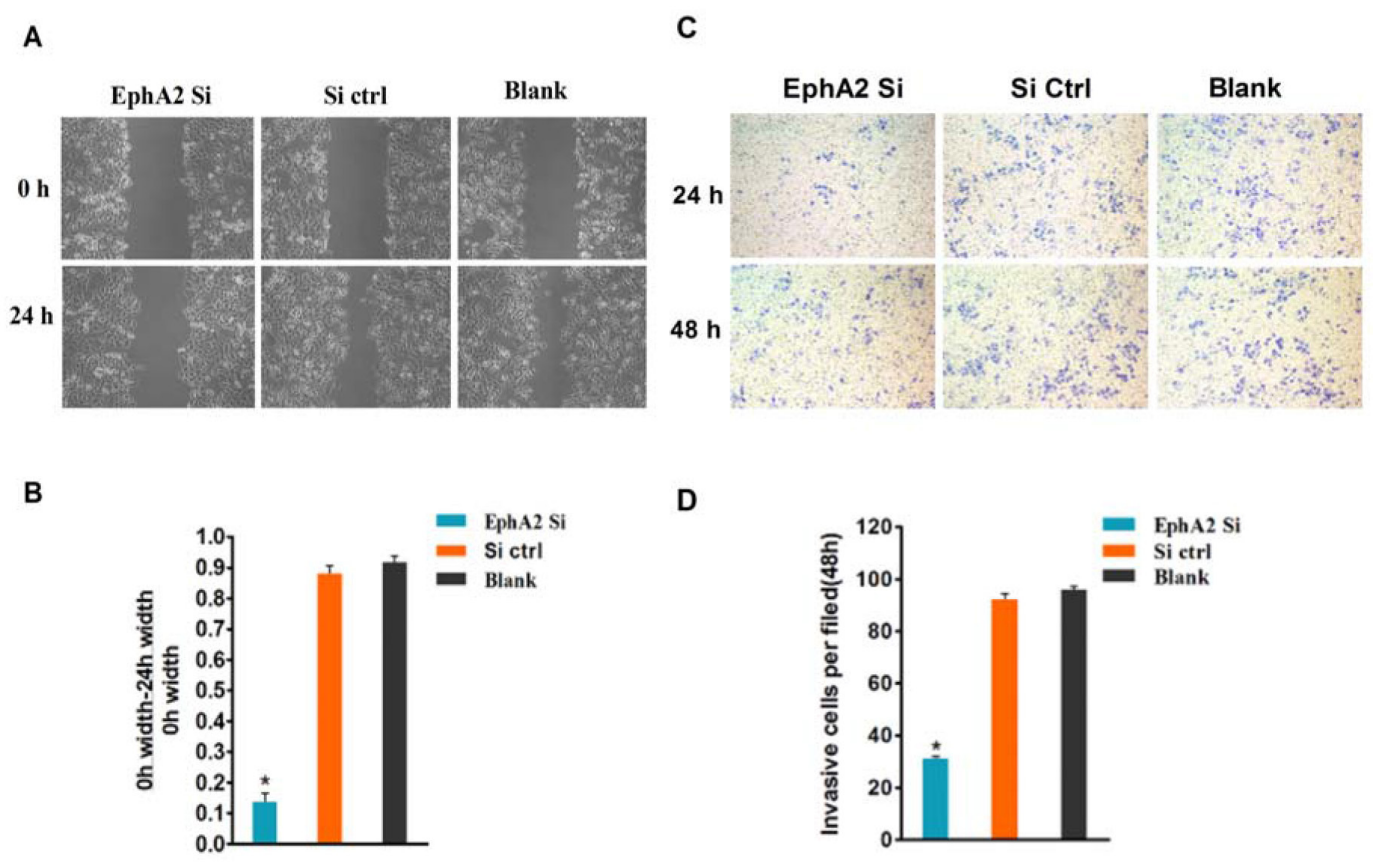
EphA2 promotes the migration and invasion of SGC-7901/L-OHP cells, and silencing of EphA2 may change their biological characteristics **(A)** Scratch wound-healing assay. Different groups of SGC-7901/L-OHP cells at two time points (0 and 24 h) are shown. **(B)** Graphical representation of the percentage of wound-healing was measured using the following formula: (0 h width of wound – 24 h width of wound) / (0 h width of wound) compared with the ‘blank’ group (* *P* < 0.05). **(C)** The invasion properties of the cells were analyzed by an invasion assay using a Matrigel-coated plate. Different groups of invasive SGC-7901/L-OHP cells at two time points (24 h and 48 h after cell seeding) are shown. **(D)** Graphical presentation of invasive cells (per field) at 48 h compared with the ‘blank’ group (*P < 0.05).

To demonstrate the effects of EphA2 on the migration and invasion of gastric cancer cells, a scratch wound-healing assay was used to validate our observations that EphA2 expression has a positive effect on cell migration ability. As shown in Figure 6A and 6B, the EphA2-silenced oxaliplatin-resistant gastric cancer cells (SGC-7901/L-OHP) migrated at a significantly reduced rate compared with the control group (*P* < 0.05). A cell invasion assay based on the Boyden chamber assay was used to provide further support for the EphA2-mediated effect on cell invasion. Images of cells that migrated through the Matrigel matrix are shown in Figure 6C and 6D. Upon inhibition of endogenous EphA2, the number of SGC-7901/L-OHP oxaliplatin-resistant gastric cancer cells migrating through the Matrigel significantly decreased compared with the control group (*P* < 0.05). These results suggest that EphA2 facilitates the migration and invasion of SGC-7901/L-OHP oxaliplatin-resistant gastric cancer cells.

### Susceptibility of oxaliplatin-resistant gastric cancer cells to L-OHP after transfection with EphA2 siRNA

**Figure 7:**
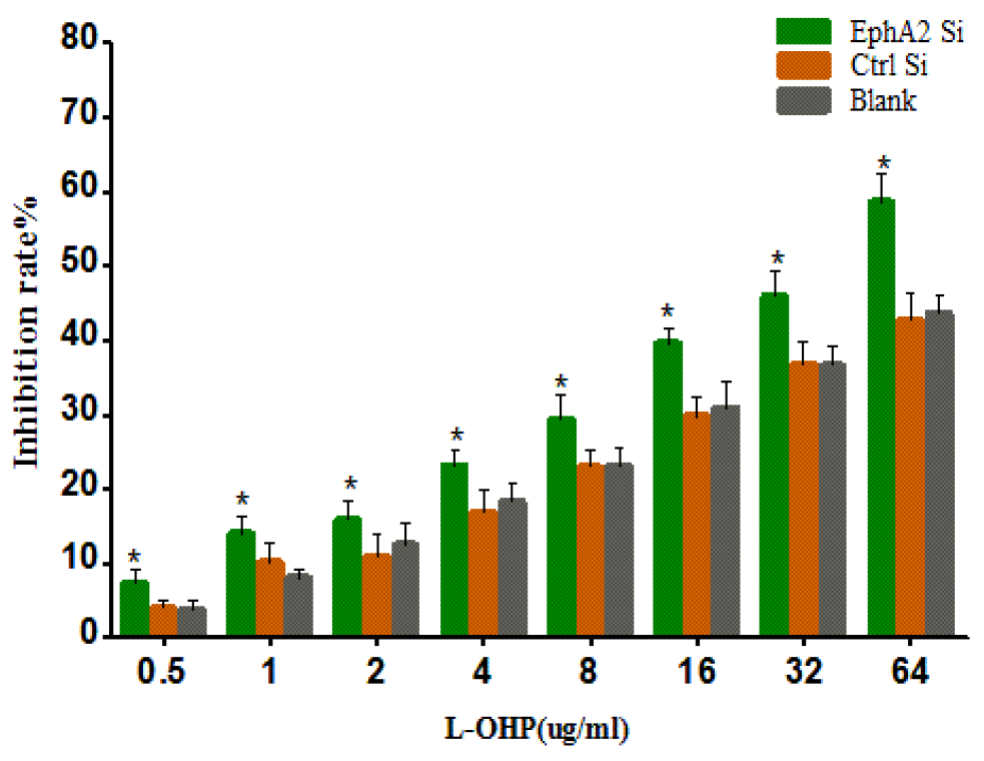
MTT assay showing that EphA2 silencing enhanced the susceptibility of SGC-7901/L-OHP cells to L-OHP (*P < 0.05)

After transfection of the oxaliplatin-resistant gastric cancer cell line SGC-7901/L-OHP with EphA2 siRNA, we tested the susceptibility of these cells to L-OHP using an MTT assay. The results demonstrated that the inhibition rates of L-OHP in each cell group increased concomitantly with an increase in L-OHP concentration. The inhibition rate of L-OHP (identical concentrations) in the EphA2 knockdown cells was significantly higher than that in control siRNA and blank cells (*P* < 0.05). These results suggest that EphA2 silencing enhances the susceptibility of SGC-7901/L-OHP cells to L-OHP (Figure 7)

## DISCUSSION

In this study, we revealed that EphA2 was overexpressed in gastric cancer patients who received chemotherapy. Furthermore, reduced EphA2 expression affected the efficacy of chemotherapy. As part of this study, the oxaliplatin-resistant human gastric cancer cell line SGC-7901/L-OHP was successfully established by induction of a continuous stepwise increase in drug concentration. This long-term exposure of the human gastric cancer cell line SGC-7901 to oxaliplatin was performed for 6 months by gradually increasing the drug dose, resulting in relatively stable drug resistance. Compared with the parental cells (SGC-7901), the drug-resistant cells exhibited long strip and fusiform morphologies with long horns stretching out from the edges of cells. The intercellular junctions of the resistant cells were relatively loose, indicating that the morphology of the oxaliplatin-resistant gastric cancer cell line SGC-7901/L-OHP was consistent with the characteristics of EMT. The drug RI of SGC-7901/L-OHP to L-OHP was 9.7 in this study. Snow [19] reported that a drug RI < 5 is indicative of low-level drug resistance, a drug RI between 5 and 15 suggests moderate-level drug resistance, and a drug RI > 15 suggests high-level drug resistance. In accordance with this proposed standard, the drug RI of SGC-7901/L-OHP to L-OHP represents moderate drug resistance, suggesting that the SGC-7901/L-OHP cell line is a reliable model of drug resistance and an ideal model for studying the mechanisms underlying drug resistance and screening for reversing agents.

The EMT is not only an early critical event involved in the induction of tumor invasion and metastasis, but is also closely related to drug resistance in tumor cells [20]. Recently, Yao [21] demonstrated that the characteristics involved in promoting distant metastasis and drug resistance in tumor cells following chemotherapy were exhibited during the process of EMT development. A series of recent studies regarding the effects of EMT on the occurrence of drug resistance in tumors demonstrated that the susceptibility of tumor cells to chemotherapeutic drugs was also significantly altered. However, in the same studies, the mesenchymal phenotype in epithelium-derived tumor cells occurred as a consequence of EMT, and mesenchymal changes in cell morphology were observed during the generation of multiple drug-resistant cell lines [22–24]. Thomson [25] found that the expression of E-cadherin in tumor tissues was extremely low after the erlotinib-resistant A549 cell line was used to inoculate nude mice. Yang [26] observed that morphological changes were present in oxaliplatin-resistant colorectal cancer cells, and loss of polarity, disappearance of intercellular junctions, presence of pseudopodia, and changes in the expression of the epithelial and mesenchymal cell markers, E-cadherin and vimentin, were also observed. In this study, quantitative real-time PCR, Western blotting and immunfluorescence analyses revealed that the EMT molecular markers, N-cadherin and Snail, were increased while E-cadherin was decreased in oxaliplatin-resistant gastric cancer cells. Further analyses revealed that the opposite effects were observed following the silencing of EphA2.

Ephs form the largest known subfamily of RTK families and the Eph proteins are involved in the transduction of multiple signal pathways and the regulation of immune function in different tissues and cells. Thus, Eph is closely related to the development and progression of tumors [27]. EphA2, a member of the RTK family, transduces extracellular signals into the cell that modulate downstream signaling networks. EphA2 upregulation is a common event in gastric cancer specimens and is closely correlated with cancer metastasis and the promotion of EMT in gastric cancer cells through activation of Wnt/β-catenin signaling [28]. Research has shown that overexpression of EphA2 in breast cancer cells promotes resistance to trastuzumab, a human monoclonal anti-HER2 antibody used for breast cancer treatment [29]. Blocking the activation of EphA2 not only prevents tumors from progressing but also increases the susceptibility of tumor cells to chemotherapy [24]. A separate study suggested that silencing EphA2 enhanced the sensitivity of lung tumors and malignant plural mesothelioma cells to Lipoplatin™ treatment [30]. Therefore, EphA2 has been suggested as a novel target for cancer treatment [31–33]. In this study, we observed that EphA2 expression significantly correlated with the expression of EMT markers in the oxaliplatin-resistant gastric cancer cell line SGC-7901/L-OHP. The suppression of EphA2 expression resulted in the inhibition of cell migration and invasion, and prevention of EMT in these cells. Thus, inhibition of EphA2 enhances the susceptibility of SGC-7901/L-OHP cells to L-OHP. The aforementioned results further validate that the reversal of oxaliplatin resistance in SGC-7901/L-OHP cells following the silencing of EphA2 results in the prevention of EMT.

In conclusion, this study reports that EphA2 functions as an EMT promoter, and that the EMT process in SGC-7901/L-OHP cells can be inhibited by EphA2 siRNA. Conversely, the susceptibility of SGC-7901/L-OHP cells to L-OHP increases concomitantly with the occurrence of EMT. These findings indicate that EphA2 promotes EMT in SGC-7901/L-OHP cell, and silencing its expression increases the susceptibility of SGC-7901/L-OHP cells to L-OHP via the EMT. Therefore, EphA2 is an excellent candidate for the development of a targeted gastric cancer therapy that will improve chemosensitivity in tumors while maintaining resistance in normal cells.

## MATERIALS AND METHODS

### Materials

Anti-EphA2, Snail, E-cadherin, and N-cadherin antibodies were purchased from Santa Cruz Biotechnology (Santa Cruz, CA, USA). Oxaliplatin was purchased from Sanofi-Aventis Pharmaceutical (Paris, France).

### Patient selection and tissue preparation

Between January 2013 and December 2016, 120 patients (including 88 male and 32 female subjects) between 28 and 76 years of age (59.2 ± 12.6 years) with gastric adenocarcinoma from the Department of General Surgery in Xiangya Hospital of Central South University (Changsha, China) were selected for this study (reference Table 2 for patient clinicopathologic features). Radical resection was performed on each of these patients. The patients received chemotherapy, but no radiotherapy prior to surgery. The study was approved by the Research Ethics Committee of Xiangya Hospital, Central South University. Cancer tissues were excised, fixed in 10% neutral-buffered formalin, and embedded in paraffin blocks.

### Evaluation of chemotherapy efficacy

According to the World Health Organization criteria for the determination of the efficacy of multiple organ tumors the subjects were divided into CR, PR, SD and PD groups. The total RR refers to CR% + PR%.

### Cell culture

The human gastric adenocarcinoma cell line SGC-7901 was obtained from the Xiangya Central Experiment Laboratory, Central South University (Changsha, China). The cells were cultured in PRMI 1640 (Hyclone, Waltham, MA, CA, USA) supplemented with 10% fetal bovine serum (Hyclone) in a humidified atmosphere of 37°C at 5% CO_2_.

### MTT assay

SGC-7901/-LOHP cells were incubated in 96-well plates at a density of 1 × 10^4^ cells per well for 24 h. Then, after the specified time points, 10 mL MTT dye (5 mg/mL; Sigma-Aldrich, St. Louis, MO, USA) was added, and the SGC-7901/-LOHP cells were incubated for another 4 h at 37°C. Next, 150 mL dimethyl sulfoxide was added to each well and the contents of the wells were mixed for an additional 10 min. Spectrometric absorbance was determined at a wavelength of 490 nm using a microplate reader (Bio-Rad, Hercules, CA, USA). Three replicates were measured for each sample.

### Plasmid construction and transfection

As previously described, the EphA2 coding sequence and EphA2 siRNA were constructed by Jikai Biomedical (Shanghai, China). A blank vector was used as a negative control [18]. A total of 5 × 10^5^ SGC-7901/L-OHP cells were seeded into each well of a 6-well plate. The SGC-7901/-LOHP cells were transfected upon reaching 80–90% confluence. Three groups of SGC-7901/-LOHP cells used in this study including untransfected cells (‘Blank Ctrl’), cells transfected with the EphA2-siRNA vector (‘EphA2 Si’) and cells transfected with the scrambled shRNA vector (‘Si Ctrl’). In the latter stages of the study, the three groups of cells were assayed to test the influence of EphA2 on EMT.

### Immunohistochemistry

The paraffin-embedded sections (4 mm thick) were cut and then deparaffinized and rehydrated. Immunohistochemical staining was performed to detect the expression of EphA2 by using the DAKO EnVision System (Dako, Glostrup, Denmark). Following proteolytic digestion, the slides were blocked with peroxidase for 30 min at room temperature with 2.5% hydrogen peroxide in methanol. The slides were subsequently incubated overnight with primary antibody at 4°C. After washing, the slices were incubated with peroxidase-labeled polymer and substrate-chromogen. Finally, sections were incubated for 5 min at room temperature in phosphate-buffered solution containing diaminobenzidine. The staining results of the targeted proteins were observed under the microscope. Negative controls were prepared by substituting the primary antibody with non-immune rabbit serum. Two independent pathologists evaluated and scored the sections following observation of 10 random visual fields for each section (double-blinded). The evaluation of the staining was graded semi-quantitatively. The expression intensity scores (0 point = 0–5%; 1 point = 6–25%; 2 points = 26–50%; 3 points = more than 50%) and positive staining cell scores (1 point = weak intensity; 2 points = moderate intensity; 3 points = strong intensity) were summed. Sum scores ≥ 3 points were considered significant overexpression and noted as positive to simplify the data analysis.

### Scratch wound-healing assay

SGC-7901/-LOHP cells were plated and grown overnight to confluence in a 6-well plate. Monolayers of SGC-7901/L-OHP cells were wounded by dragging a pipette tip across the monolayer. SGC-7901/L-OHP cells were washed to remove cellular debris and allowed to migrate for 24 h. Images were taken at 0 and 24 h after wounding under the inverted microscope.

### Cell invasion assay

Transwell invasion assays were performed in 24-well 8 mm pore size transwell plates according to the manufacturer's instructions (Corning, New York, USA). The bottom of each transwell chamber was coated with BD Matrigel Basement Membrane Matrix (BD Biosciences, San Diego, CA, USA). The upper chamber was filled with 1 × 10^5^ cells in RPMI 1640 containing 5% fetal bovine serum, and the lower chamber was filled with RPMI 1640 containing 25% fetal bovine serum (this served as a chemoattractant). After the chambers were incubated for 24 h or 48 h at 37°C, non-invading cells on the upper side of the chamber were removed from the surface of the membrane by scrubbing, and invading cells on the lower surface of the membrane were fixed in methanol, mounted, and dried. The number of cells invading through the matrigel was counted by a technician blinded to the experimental settings. The cells were counted in four randomly selected microscopic fields for each filter. The test was repeated three times.

### Immunfluorescence

SGC-7901/L-OHP cells were grown in dishes and fixed for 30 min in 4% paraformaldehyde. The SGC-7901/L-OHP cells were permeabilized with 0.04% Triton X-100 for 10 min. The primary antibody, diluted in phosphate-buffered solution/5% bovine serum albumin, was applied for 1 h at 37°C. Following phosphate-buffered solution washing steps, the appropriate Alexa Fluor 488-linked secondary antibody (Invitrogen, Carlsbad, CA, USA) was applied for 30 min at room temperature. Controls were performed omitting the primary antibody and with the corresponding immunoglobulin G fraction as the primary antibody. SGC-7901/L-OHP cells were counterstained with SGC-7901/L-OHP cells were coun, and images were viewed under a fluorescence microscope.

### Real-time reverse transcriptase–PCR

Total RNA was extracted using the Trizol (Invitrogen, Carlsbad, CA, USA). following the manufacturer's instructions. The cDNA was synthesized using 1 mg of total RNA and TaqMan Reverse Transcription Reagents (Applied Biosystems, Foster City, CA, USA). The primers for real-time PCR were listed as Supplementary Table 1. The relative levels of target gene mRNA were expressed as the ratio of target to 18S and calculated from the standard curve as directed. All of the reported results are the average ratios of three different independent experiments.

### Western blotting

The cells were grown to confluence and lysed in lysis buffer Equal amounts of protein were loaded and resolved on 10% SDS-PAGE ( Bio-Rad Laboratories, Hercules, CA, USA) Whole-cell extracts were prepared using 0.14 M NaCl, 0.2 M triethanolamine, 0.2% sodium deoxycholate, and 0.5% Nonidet P-40 supplemented with a protease inhibitor (Sigma). A total of 40 μg protein was loaded into each well. Proteins were electrotransferred onto membranes that were subsequently blocked and incubated overnight at 4°C with primary antibody. Next, the membranes were incubated with secondary antibody for 1 h. Then, the bands were visualized and quantitated using the ECL Advance Detection System (Amersham Biosciences, Piscataway, NJ, USA).

### Statistical analysis

The protein expression levels were compared with χ^2^ tests. Continuous variables were evaluated using the unpaired Student's *t*-test. Bivariate correlations between study variables were calculated by Spearman's rank correlation coefficients. Measurement data were expressed as mean ± standard deviation (SD). Statistical analyses were performed with SPSS 19.0 software. *P* < 0.05 was considered statistically significant.

## SUPPLEMENTARY MATERIALS AND TABLES


