# Supplementary material for: EphA2 affects the sensitivity of oxaliplatin by inducing EMT in oxaliplatin-resistant gastric cancer cells
Source: Oncotarget. 2017 May 24;8(29):47998–8011. doi: 10.18632/oncotarget.18208 (PMC5564621; doi:10.18632/oncotarget.18208)
Supplement: Supplementary file 1 [file oncotarget-08-47998-s001.pdf]

EphA2 affects the sensitivity of oxaliplatin by inducing EMT in oxaliplatin-resistant gastric cancer cells

SUPPLEMENTARY INFORMATION

Supplementary Table 1: EphA2, E-cadherin, N-cadherin, snail and 18S primers

	Primers	Nucleic acid sequence(5'-3')	The product size(bp)
EphA2	Right	CTGGCTCACACACCCGTATGGC	209
	Left	CGCCACCAGGGAAGCTGTTG	
E-cadherin	Right	CAACGACCCAACCCAAGAA	271
	Left	CCGAAGAAACAGCAAGAGCA	
N-cadherin	Right	TTGGTTTGGGGAGGGAGA	234
	Left	CTGGGGTCAGAGGTGTATCATTT	
Snail	Right	TGCTCCACAAGCACCAAGA	228
	Left	GCAGAGGACACAGAACCAGAAA	
18S	Right	AAATAGCCTTTGCCATCACTGC	181
	Left	GTTCAAGAACCAGTCTGGGATC	
